# Targeted DNA Sequencing Detects Mutations Related to Susceptibility among Familial Non-medullary Thyroid Cancer

**DOI:** 10.1038/srep16129

**Published:** 2015-11-04

**Authors:** Yang Yu, Li Dong, Dapeng Li, Shaokun Chuai, Zhigang Wu, Xiangqian Zheng, Yanan Cheng, Lei Han, Jinpu Yu, Ming Gao

**Affiliations:** 1 Department of Thyroid and Neck Tumor, Tianjin Medical University Cancer Institute and Hospital, National Clinical Research Center for Cancer, Huanhuxi Road, Ti-Yuan-Bei, Hexi District, Tianjin 300060, China; 2 Cancer Molecular Diagnosis Core, Tianjin Medical University Cancer Institute and Hospital, National Clinical Research Center for Cancer, Huanhuxi Road, Ti-Yuan-Bei, Hexi District, Tianjin 300060, China; 3 Key Laboratory of Cancer Prevention and Therapy, Tianjin, Huanhuxi Road, Ti-Yuan-Bei, Hexi District, Tianjin 300060, China; 4 Guangzhou Burning Rock Biotech, Guangzhou International Biotech Island (GIBI), Guangzhou 510300, China

## Abstract

Some studies have demonstrated that familial non-medullary thyroid cancer (FNMTC) has a more aggressive clinical behavior compared to sporadic NMTC (SNMTC). However, FNMTC is difficult to differentiate from SNMTC by the morphology and immunohistochemistry. Although genes responsible for FNMTC were unclear, screening for rare germline mutations on known important tumor suppressor genes might offer more insights on predicting susceptibility to FNMTC. Here, a customized panel was designed to capture all exons of 31 cancer susceptive genes possibly related to FNMTC. Using next-generation sequencing we performed deep sequencing to achieve 500× coverage of the targeted regions. At the end 45 variants were identified in 29 of 47 familial patients and 6 of 16 sporadic patients. Notably, several germline mutations were found matching between paired FNMTC patients from the same family, including APC L292F and A2778S, BRAF D22N, MSH6 G355S and A36V, MSH2 L719F, MEN1 G508D, BRCA1 SS955S, BRCA2 G2508S, and a GNAS inframe insertion. We demonstrated a novel approach to help diagnose and elucidate the genetic cause of the FNMTC patients, and assess whether their family members are exposed to a higher genetic risk. The findings would also provide insights on monitoring the potential second cancers for thyroid cancer patients.

Thyroid cancer is the most common malignancy of endocrine organs, and its prevalence is increasing rapidly. Non-medullary thyroid cancer (NMTC) arising from the follicular thyroid epithelium cells accounts for more than 95% of all thyroid cancers^1^. About 5%–10% of NMTC patients have reported familial history, therefore named familial NMTC (FNMTC)^2^,^3^,^4^.

FNMTC is defined by the diagnosis of two or more first degree relatives affected by differentiated thyroid cancer of follicular cell origin. FNMTC can be divided into two groups based on clinical characteristics. The first includes familial tumor syndromes characterized by a preponderance of non-thyroidal tumors, for example, familial adenomatous polyposis (FAP), Gardner’s syndrome, Cowden’s disease and so on. The second group includes familial syndromes characterized by a preponderance of NMTC. Histologically, FNMTC includes papillary thyroid carcinoma (PTC), follicular thyroid carcinoma (FTC), and anaplastic thyroid carcinoma. However, FNMTC is difficult to differentiate from sporadic non-medullary thyroid cancer (SNMTC) because FNMTC is usually indistinguishable from sporadic follicular cell cancers either by morphological examination or immunohistochemical assay, which means that FNMTC cannot be diagnosed until at least one of the patient’s first degree relatives is affected by NMTC.

Some studies have demonstrated that compared to SNMTCs, FNMTCs have a more aggressive clinical behavior, as well as a worse prognosis^5^,^6^,^7^,^8^,^9^. Patients with FNMTC have an increased risk of multifocal disease, local invasion, increased local or regional recurrence, lymph node metastases, and intraglandular dissemination. Comparing the first and the second generation of FNMTC patients, the off-springs usually show an earlier age at disease onset and have more aggressive disease when compared with their parents^10^. Although the prognosis of FNMTC is poor compared to SNMTC, standardized treatment performed at early stage of disease is considerably effective to reduce the relapse and metastasis after surgery. However, since there has not been a sensitive and specific experimental approach available in clinic to screen for high genetic risks related to FNMTC, it’s difficult to identify susceptible FNMTC patients at an early stage. This could lead to insufficient treatment in clinic and higher risk of post-operation relapse for those FNMTC patients.

Reviews of various genetic studies suggest that inheritance of FNMTC is autosomal dominant and that the penetrance is incomplete^9^,^11^,^12^,^13^,^14^,^15^,^16^. In 1997, Bignell^16^ evaluated the contribution of MNG1 to FNMTC, studied 37 NMTC families and found that a small group of patients could be attributed to MNG1 (14q32). Canzian^15^ investigated a French pedigree with multiple cases of multinodular goiter and NMTC and reported the mapping of the responsible gene, named “TCO”. TCO was mapped to chromosome 19p13.2 by linkage analysis with a whole genome panel of microsatellite markers. McKay’s study^11^ found the existence of a susceptibility locus for FNMTC on chromosome 2q21 (NMTC1). Malchoff^13^ demonstrated that the incidence of an America family (five PTC and two papillary renal neoplasia, PRN) was linked to 1q21 (FPTC/PRN). In one study, the authors evaluated the roles of MNG1, TCO1, FPTC/PTEN, TSHR, and TRKA in FNMTC, and carried out a comprehensive mutation and linkage analysis of these genes in 22 families^12^. One family was linked to chromosome 19q13.2, confirming that TCO1 underlies a subset of familial non-medullary thyroid cancer. However, none of the LOD scores in this study actually achieved statistical significance, including for TCO1. Lack of successful identification of significant loci from these studies indicates that the genetic causality model for FNMTC might be complex. On the other hand, there are tumor suppressor genes whose germline mutations are known to correlate with some NMTC-related familial tumor syndromes, e.g. PTEN with Cowden Syndrome and WRN with Werner Syndrome^17^,^18^. Therefore, instead of the classical linkage analysis approach, screening for rare germline mutations on known important tumor suppressor genes might offer more insights on predicting susceptibility to FNMTC.

The key challenge on tumor suppressor genes mutation profiling is that the whole exons have to be sequenced, since most loss-of-function mutations do not demonstrate any “hot spot” patterns. Next-generation sequencing (NGS) offers simultaneous sequencing of thousands to millions of short nucleic acid sequences and may offer a cost-effective approach for parallel detection of multiple genetic alterations. It provides a clear advantage over the conventional sequencing technique, such as Sanger sequencing, by allowing simultaneous analysis of large regions of the genome and offering high sensitivity of mutation detection and quantitative assessment of mutant alleles. In this study, we evaluated capture-based targeted DNA sequencing as a new approach for testing a broad spectrum of point mutations (SNVs) and short insertion-deletions (Indels) possibly related to FNMTC. Targeted sequencing enables deep coverage of the interested genomic regions only with affordable cost per patient. Here, we assess the feasibility of such an approach in a NMTC-patient cohort and found a few mutational loci in FNMTC patients that haven’t been reported before.

## Results

### Quality assessment of the targeted sequencing data

**Figure 1:**
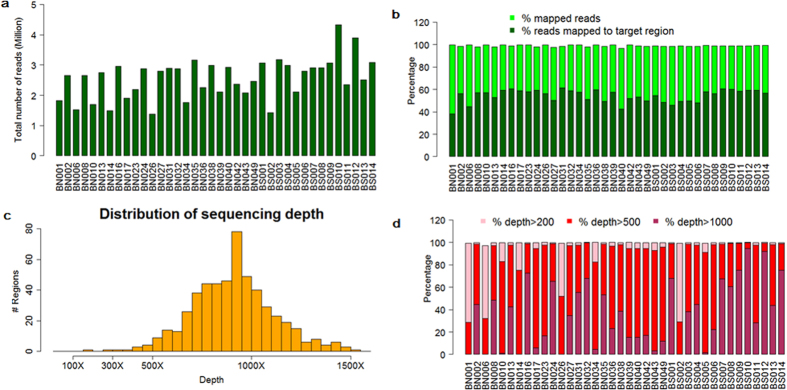
Quality assessment of the targeted sequencing data among 38 samples in first batch. (**a**) Total number of reads in every sample (with an average of 2.46 million reads for each sample). (**b**) Percentage of all mapped reads and percentage of reads mapped to target regions for every sample. (**c**) The average distribution of sequencing depth over regions across all samples (the average coverage depth was well-distributed among regions, with a minimum of 157× and a maximum of 1505×). (**d**) The coverage depth in each sample (all samples have greater than 96% of all regions with coverage depth greater than 200×).

We performed deep sequencing with the FNMTC susceptibility panel on two batches of samples to achieve average 500× coverage of the targeted regions. Among the 38 samples in the first batch we profiled, we obtained an average of 2.46 million reads for each sample (Fig. 1a). On average 98.9% of all reads can be mapped back to the genome and 54.3% of all reads are mapped to our designed target regions (Fig. 1b). This indicates a high capture efficiency of the probes. We then assessed the distribution of the coverage within the target regions. Figure 1c plots the histogram of the average coverage depth among the 38 samples across all target regions. The average coverage depth appears to be normally distributed, with a minimum of 157× and a maximum of 1505×. Figure 1d shows the coverage depth in each sample, where all samples have greater than 96% of all regions with coverage depth greater than 200×.

**Figure 2:**
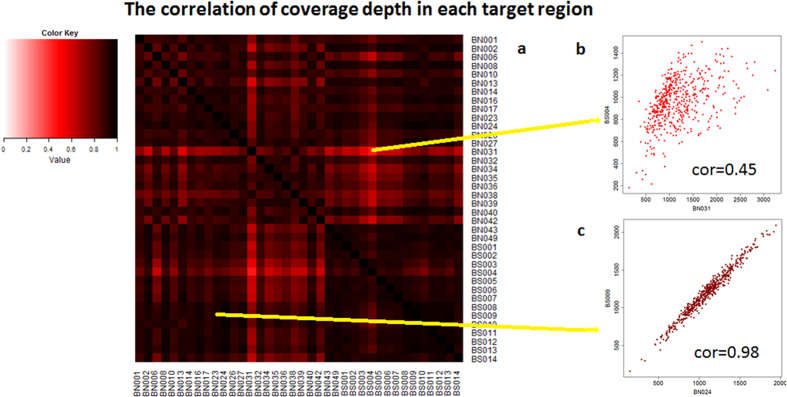
The correlation of coverage depth in each target region between 38 samples in first batch. (**a**) A heatmap of such correlation between each pair of samples. (**b**) The scatterplot of coverage depth across all regions in the least correlated pair of samples. (**c**) The scatterplot of coverage depth across all regions in the most correlated pair of samples.

We then assessed the repeatability of probe capturing efficiency by measuring the correlation of coverage depth in each target region between different samples. Figure 2a shows a heatmap of such correlation between each pair of samples. Most pairs have a correlation coefficient greater than 0.8, indicating excellent repeatability of capturing efficiency of probes across different regions. The scatterplots of coverage depth across all regions between the most and least correlated pairs of samples are also shown (Fig. 2b,c). Sequencing data quality and region coverage reproducibility is similar among the second batch of samples (see Supplementary Fig. S1–2 online).

### Germline mutation identification

**Figure 3:**
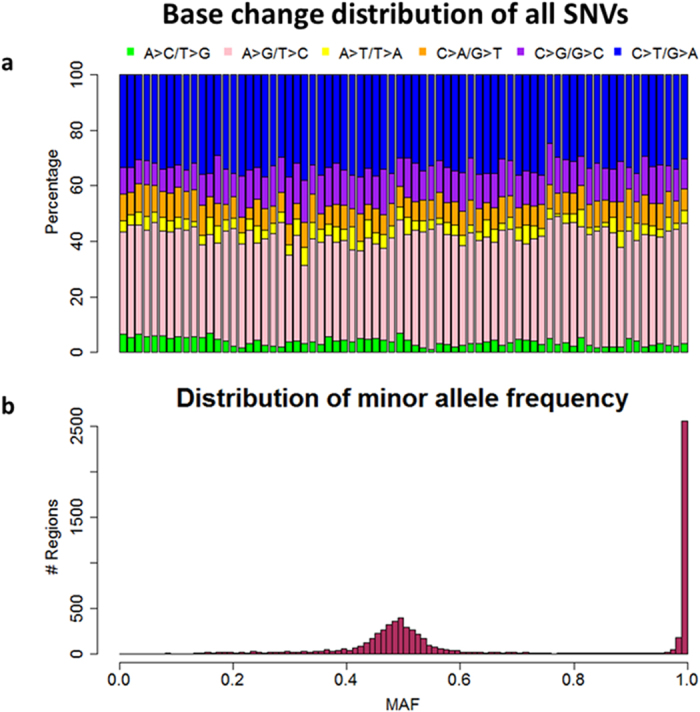
Base change distribution of all SNVs and distribution of MAF. (**a**) Base substitution distribution of all single-nucleotide variants (SNVs) across all samples. (**b**) The distribution of the minor allele frequencies (MAF) across all identified variants.

We applied GATK 3.2 for genotyping the two batches of samples and identified 8462 single-nucleotide variants (SNVs) and 1699 insertion/deletion (INDELs) overall. The base substitution pattern is highly consistent across all samples (Fig. 3a), with an average transition-transversion (ti/tv) ratio of 2.55, similar to previously reported ti/tv ratio among exome regions. We then plotted the distribution of the minor allele frequencies (MAF) across all identified variants (Fig. 3b). The MAF demonstrates a clear bi-modal distribution, peaking at 0.5 and 1, a distribution expected for germline variants.

**Figure 4:**
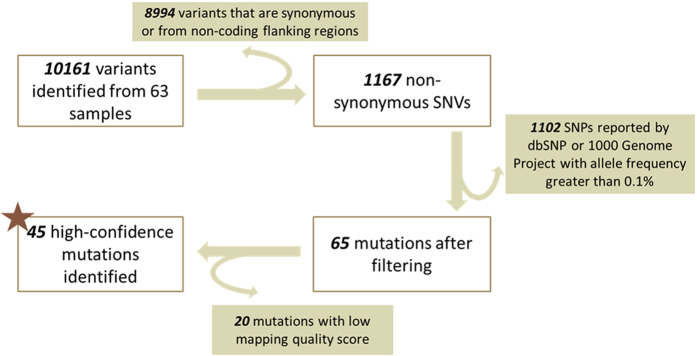
Process of mutation filtering. Among 10161 variants identified from 63 samples, there were 8994 variants that were synonymous or from non-coding flanking regions, 1102 SNPs reported by dbSNP or 1000 Genome Project with allele frequency greater than 0.1%, 20 mutations with low mapping quality score. After filtering out above variants 45 variants were identified in the end and listed in Table 1, including 38 SNVs and 7 indels. The MAF of these SNVs ranges from 43.4% to 60.0%, indicating that all of them are heterozygous germline mutations.

In order to identify clinically meaningful germline variants, we applied filters to pick out non-synonymous high-confidence variants that were not reported previously in dbSNP or 1000 Genome Project as a common SNP (Fig. 4). Through the filtering steps, 45 variants were identified in the end and listed in Table 1, including 38 SNVs, one short 4-bp frame-shift deletion, one splice donor variant and five in-frame insertions or deletions. The MAF of these SNVs ranges from 43.4% to 60.0%, indicating that all of them are heterozygous germline mutations.

We annotated all 45 variants with both the ExAC and ClinVar databases. Twenty-three out of the 37 variants (62%) from the familial group and 2 out of the 8 variants (25%) from the sporadic group are archived in the ExAC database. We examined the prevalence of all variants among both the whole ExAC population and the East Asian population only. Most archived variants have lower than 0.0001 minor allele frequency in both populations. Interestingly, 19 out of all 25 (78%) ExAC-archived variants show a greater prevalence among East Asian population than among the whole ExAC population. Twenty out of the 37 variants (54%) and 2 out of the 8 variants (25%) have at least one previous submission in the ClinVar databases. However, most of them have unclear clinical interpretation offered by ClinVar. None of the variants are archived as clearly pathogenic. One variant, MSH6 L1358fs, even though appearing to be a loss-of-function mutation, is archived as “likely benign”, probably because it is located at the very end of the MSH6 protein.

Since we could only find limited information about the detected variants from ExAC and ClinVar, in order to elucidate their possible impact on cancer susceptibility, we further annotated all variants with eight different tools, including SIFT, Polyphen2, LRT, MutationTaster, MutationAssessor, FATHMM, MetaSVM, and MetaLR, to predict the impact of the variants on protein function (Table 1; More details are available in Supplementary Table S1 online). Out of all 45 variants detected, 14 of them were predicted to be damaging or deleterious by at least half of the predicting algorithms. Most importantly, three matching variants within FNMTC families (BRCA2 G2508S, MSH2 L719F, and APC A2778S) were predicted to be damaging. The BRCA2 variant was also predicted to be highly likely to be damaging by GVGD (Class C55), further indicating possible contribution of these variants to the patients’ susceptibility (Table 1; More details are available in Supplementary Table S1 online).

**Figure 5:**
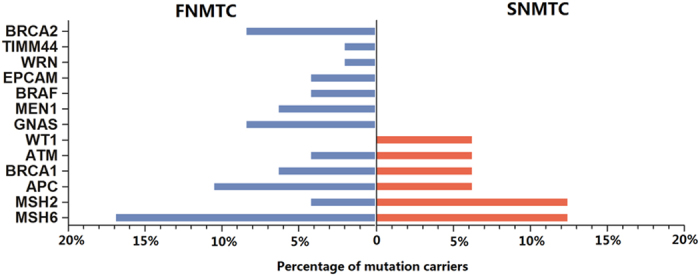
The distribution of mutational genes between the two groups. (12 mutational genes were detected in FNMTC patients and 6 in SNMTC cases; APC, BRCA1, BRCA2, GNAS, MSH2 and MSH6 were common genes detected in the two groups of patients).

**Figure 6:**
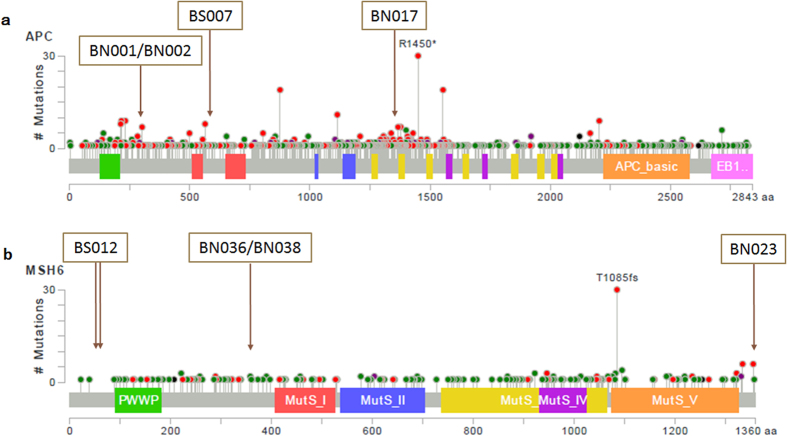
Mutations detected in APC and MSH6 are overlaid on mutation distribution diagrams from TCGA. (**a**) APC. (**b**) MSH6.

**Figure 7:**
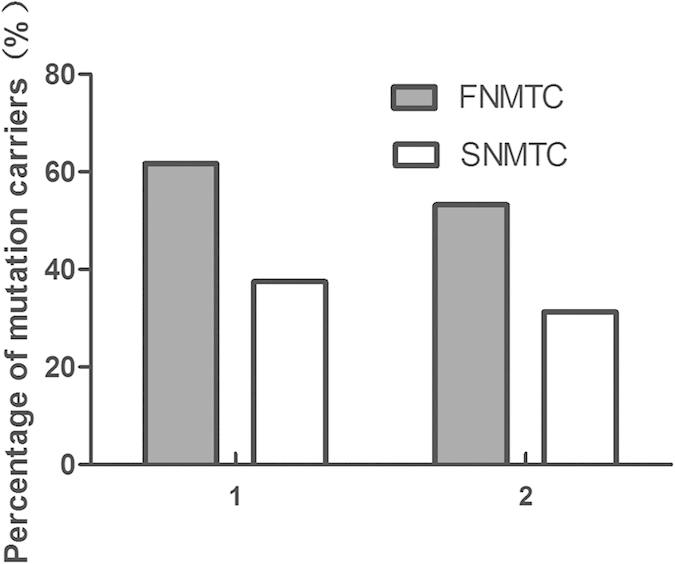
The rate of germline mutation carriers. (“1” represents the rate of any germline mutation carriers in FNMTC group and in SNMTC group (61.7% vs. 37.5%); “2” represents the rate of mutations of eight genes with recurrent germline mutations detected (53.2% in FNMTC vs. 31.3% in SNMTC)).

Mutations were detected among 13 out of the 31 genes profiled, including APC, MSH2, MSH6, ATM, BRAF, BRCA1, BRCA2, EPCAM, GNAS, MEN1, TIMM44, WRN and WT1. The distribution of mutational genes between the two groups was shown in Fig. 5. Twelve mutational genes were detected in FNMTC patients and six in SNMTC cases. The mutational spectrum of FNMTC was wider than that of SNMTC. The six most common genes detected in the two groups of patients were APC, BRCA1, BRCA2, GNAS, MSH2 and MSH6. Mutations detected in APC and MSH6 are overlaid on mutation distribution diagrams from TCGA (Fig. 6). Ten patients (8 FNMTC and 2 SNMTC) carry two germline mutations among all genes profiled, while all other patients carry one or none. We then compared the percentage of any germline mutation carriers between the two groups. Twenty-nine out of 47 (61.7%) among the familial group and 6 out of 16 (37.5%) among the sporadic group display a marginal but not statistically significant difference (OR = 2.64; p-value = 0.14), largely due to the limited sample size (Fig. 7). Notably, ten germline mutations from eight genes were found matching between paired FNMTC patients from the same family, including APC L292F and A2778S, BRAF D22N, MSH6 G355S and A36V, MSH2 L719F, MEN1 G508D, BRCA1 SS955S, BRCA2 G2508S, and a GNAS inframe insertion. The mutation percentage of these eight genes in FNMTC group was 53.2%, while 31.3% in SNMTC group (OR = 2.46; P = 0.16). It’s plausible that some of these variants might have contributed to these patients’ susceptibility to thyroid cancer.

### Association of germline mutations and clinical-pathological characteristics

The clinical-pathological information of 61 patients in this research was collected and based on sequencing results, patients were divided into two groups: mutation positive and mutation negative. We compared the clinical features of mutation positive patients with that of negative patients (Table 2). There was a trend for a younger age in the positive group (mean 43.26 ± 9.66 vs. 47.19 ± 13.02, P = 0.18). No statistically significant differences were identified between the two groups on other factors, including female/male ratio (28: 7 vs. 20: 6, p = 0.77), tumor size (1.05 ± 0.50 vs. 1.01 ± 0.47, p = 0.73), the presence of multifocal disease (45.7% vs. 42.3%, p = 0.79) and the presence of bilateral disease (34.3% vs. 34.6%, p = 0.98). Interestingly, we found that central lymph nodes involvement occurred more frequently in the mutation-positive group than in the negative group (68.6% vs. 30.8%, p = 0.003), even though the treatment approach, such as extension of surgery, displayed no statistically significant difference between the two groups of patients.

## Discussion

In this study, we developed a new approach for identifying potential genetic risk factors for FNMTC using next-generation sequencing. It uses a small amount of DNA and tests for a broad range of point mutations and indels simultaneously with high accuracy and sensitivity. The panel includes whole exon regions from 31 genes and one intronic region from RET for potential RET fusion profiling. Sequencing data showed comprehensive and evenly distributed coverage among different regions. With an average of ~500× coverage depth, more than 99% of all regions were covered by more than 200 reads. Furthermore, the high coverage distribution correlation between different batches of samples indicates a great reproducibility. Given such distribution, since germline mutations mostly possess an allele frequency around 50% or 100%, future application of this NGS panel for germline mutation detection would only need an average coverage depth of 50–100×, equalizing 17 MB sequencing data per sample, which would be very affordable for clinical application.

Our study has identified 37 variants among 29 (61.7%) NMTC patients with strong familial history and 8 variants among 6 (37.5%) NMTC patients without apparent familial history. Although statistical significance was not reached (p = 0.14), a notable OR of 2.64 was observed. It would have reached statistical significance if the sample size increased by 50% and prevalence in each group remained the same. It should also be noted that the co-occurrence pattern in many families with multiple NMTC cases might be attributed to environmental factors but not genetic risks.

Interestingly, when ignoring the familial pattern and simply comparing the clinical features between mutation positive patients with mutation negative patients, we found that the former group showed a trend for younger age and more frequent central lymph nodes involvement though they had no different in tumor size. This is consistent with previous studies, which suggested patients with FNMTC have an increased risk of lymph node metastases^5^,^19^,^20^. However, lymph node metastasis in central compartment could not be identified by preoperative examination easily. Therefore, central compartment neck dissection is needed for patients with such germline mutation or family history of thyroid cancer, at least central lymph nodes ipsilateral to the lesion should be removed.

The six commonly mutated genes detected in this study were APC, BRCA1, BRCA2, GNAS, MSH2 and MSH6. There are five patients in FNMTC group with APC mutations, four patients of whom belong to two families, indicating that the shared variants of APC may be associated with the incidence of NMTC. Defects in this gene cause familial adenomatous polyposis (FAP), an autosomal dominant pre-malignant disease that usually progresses to malignancy. An association between follicular adenoma and FAP has been described^21^,^22^,^23^. It had been reported that the cribriform-morular variant of papillary thyroid carcinoma was frequently seen in patients with FAP^24^ and thirty percent of thyroid carcinomas are diagnosed 4–12 years before the development of polyposis coli^21^. Although the siblings carrying APC mutation are follicular variant of papillary thyroid carcinoma in this study, we could not confirm whether they have adenomatous polyposis. Therefore regular colonic examination is recommended for these patients. In another family, the siblings have the same variant of BRAF D22N, different from the well-known BRAF V600E variant that is highly prevalent in papillary thyroid carcinoma. The highly mutated genes (KRAS, HRAS, NRAS, etc.) in somatic cells of thyroid cancer were rarely observed to mutate in patients’ blood in our study.

Matching mutations within family in MSH6 or MSH2 were detected in three other families. MSH6 and MSH2, together with MLH1 and PMS2, belong to DNA mismatch repair (MMR) genes, preventing mutational events through correction of mismatched bases during DNA replication. Germline mutations in MMR genes can give rise to the autosomal dominant condition, Lynch Syndrome (LS), previously referred to as hereditary nonpolyposis colon cancer (HNPCC). LS was characterized by increased lifetime risks for colorectal (40–80%), endometrial (25–60%), ovarian (4–24%), and gastric (1–13%) cancers^25^,^26^. During our follow-up with the tested patients, patient BN040, who carries the same MSH2 L719F mutation as her sibling, reported suffering from frequent diarrhea (3–4 times per day), and was recommended for further examination. We also learned that within family BN10, from which two family members were detected with MSH6 G355S in our study, four out of all five sisters suffer from thyroid cancer. Rein^27^ reported a 44-year-old woman from a Lynch syndrome, Amsterdam positive family who was referred for DNA testing. She had a recent history of a colorectal adenoma and an undifferentiated carcinoma of her thyroid and was shown to carry the truncating MSH2 mutation that was known to segregate in her family. Traditionally, thyroid cancer is not considered to be part of the Lynch syndrome tumour spectrum. However, Rein’s study demonstrated that the patient’s undifferentiated thyroid carcinoma showed complete loss of immunohistochemical expression of the MSH2 and MSH6 protein. In addition, Pande’s findings^28^ confirmed that a significant number of Lynch syndrome patients can present with thyroid carcinoma or other types of tumors as primary tumors that are not part of the Lynch syndrome spectrum. Our sequencing results showed germline mutations of some MMR genes detected in 37.1% (13/35) of all NMTC patients with a mutation. Therefore, the causality of MMR gene mutations on thyroid carcinoma calls for further investigation. Screening of colonic polyposis by total colonoscopy should be recommended for thyroid carcinoma patients carrying MMR gene mutation. Studies had demonstrated that annual surveillance of colon for MMR mutations patients would reduce the incidence of colon cancer and mortality^29^,^30^,^31^. Women carrying these germline mutations have dramatically elevated rates of gynecological cancer compared to women in the general population. They face a 40–60% lifetime risk of endometrial cancer and a 10–12% lifetime risk of ovarian cancer, compared to the general population risks of 3% and 1.4%, respectively^32^,^33^,^34^. Some authors recommend annual surveillance in this high-risk group of mutation carriers, including transvaginal ultrasonography, tumor marker CA125 blood tests and/or endometrial biopsy^35^,^36^. Schmeler’s findings^37^ suggest that prophylactic hysterectomy with bilateral salpingo-oophorectomy is an effective strategy for preventing endometrial and ovarian cancer in women with germline mutations related to the Lynch syndrome.

Previous studies^38^,^39^,^40^,^41^,^42^ have indicated that patients with thyroid cancer have an increased risk of developing a second cancer in all sites examined including salivary gland, kidney, prostate, skin, breast, brain, myeloma, leukemia, and non-Hodgkin lymphoma, compared to the general population. Fallah^43^ studied lifetime cumulative risk of thyroid cancer (CRTC) in 63495 first degree relatives of 11206 NMTC patients, Family history of PTC increased risk of non-thyroid endocrine cancers (such as parathyroid cancer) and non-melanoma skin cancer up to about two-fold in both sexes and kidney (1.4-fold) and prostate (1.2-fold) cancer in men. An additional advantage of this panel is that it might help to forecast the risk and the type of second tumor after diagnosis of primary thyroid cancer from the germline genetic variant that patient carries. For example, mutations in ATM, BRCA1 and BRCA2 increase the risk of breast cancer and ovarian cancer, while WT1 is commonly expressed in ovarian serous carcinomas and endometrial serous carcinoma and considered a diagnostic marker of these tumors^40^. MEN1 and RET are associated with multiple endocrine neoplasia type I and multiple endocrine neoplasia type II, respectively. During our follow-up with mutation-positive patients from this study, one SNMTC patient BS012 has most recently developed a 3-cm lump in her breast. She is undergoing further examination for diagnosis.

In summary, we demonstrated the feasibility and practicability of the application of next generation sequencing in screening for germline mutations on cancer-related genes among FNMTC patients. This method provides a novel approach for us to help diagnose and elucidate the genetic cause of the FNMTC patients, and assess whether their family members are exposed to a higher genetic risk. The findings would also provide insights on monitoring the potential second cancers for patients affected with thyroid cancer.

## Methods and Materials

### Ethics statement

Peripheral blood of all patients was obtained from Tumor Tissue Banking Facility of Tianjin Medical University Cancer Institute and Hospital and all participants provided written informed consent. This study was performed in accordance with the approved guidelines of Tianjin Medical University Cancer Institute and Hospital’s Ethics Committee.

### Patients

FNMTC is defined by the diagnosis of two or more first-degree relatives affected by differentiated thyroid cancer of follicular cell origin. Therefore, in the present work, the criteria for eligibility of the FNMTC families were that two (or more) first-degree family members had to be affected with NMTC. In this study, total of 63 NMTC patients were recruited, including 47 cases with an apparent family history of thyroid cancer and 16 cases with no family history. The task of sequencing and analysis was completed in two times. In the first batch of samples, 24 FNMTC patients from 12 unrelated families and 14 patients with SNMTC were included. The ratio of female and male was 3:1 and the average age was 46 years. There were 23 FNMTC patients from 10 families and 2 SNMTC patients in the second batch which was the complement and verification for the first batch of samples. The ratio of female and male was 5.3:1 and the average age was 43 years. Histological types of all patients were papillary thyroid carcinoma. In addition, the clinic-pathological characteristics, including tumor size, extension of surgery, lymph node metastasis and extra-thyroidal extension, were compared between patients with or without actionable mutations. But the operations of two cases were not performed in our hospital, so the clinical-pathological information of them was absent.

### Design of Gene Chip

One study suggested that premenopausal breast carcinoma may occur with a greater frequency than expected in NMTC patients^44^. It has been hypothesized that breast carcinoma is a side effect of 131I therapy for PTC^45^. Alternatively, breast and thyroid carcinoma, which both arise from an epithelial cell type, may share a common susceptibility factor, gene or otherwise. Consistent with this hypothesis, studies of malignancies in first-degree relatives of NMTC patients showed a greater frequency of breast cancer than expected among first-degree relatives of FNMTC patients^46^,^47^. Other malignancies potentially associated with PTC include kidney, ovarian, and right-sided colon cancer^46^. In order to expand the screening of susceptibility genes, the panel we designed includes candidate susceptibility genes and loci for hereditary thyroid cancer: TIMM44, DNMT1, SMARCA4, RET, MEN1 and possible susceptibility genes for breast cancer and ovarian cancer, as well as other previously known familial cancer or cancer-related syndromes: BRCA1, BRCA2, TP53, PTEN, STK11, APC, CDH1, MITF, VHL, WT1, SMAD4, MUTYH, MLH1, MSH2, MSH6, PMS2, EPCAM, ATM, GNAS1, PRKAR1A, WRN, which may also relate to the incidence of thyroid cancer.

In addition, recent studies of rare hereditary lung cancer showed that carrying known disease-relevant somatic mutations (e.g. EGFR T790M) as germline might be another important mechanism of hereditary cancer syndromes^48^,^49^. Therefore, our panel also included important genes with somatic mutations found previously in thyroid cancer, multiple endocrine adenoma, parathyroid carcinoma and pheochromocytoma, for example, RET fusion, BRAF, KRAS, HRAS, NRAS, PTEN, and PIK3CA. Overall, the panel included whole exon regions from 31 genes and one intronic region from RET for potential RET fusion profiling.

### Targeted DNA sequencing

To design the capture probe baits and prepare the SureSelect reagents, 170 kb human genomic sequence from 526 target regions were submitted to the Agilent eArray platform and manufactured by Agilent.

Peripheral blood was collected from 47 FNMTCs patients and 16 SNMTCs respectively. DNA was extracted from peripheral blood leukocytes using standard protocols. DNA of the 63 samples were extracted (QIAamp DNA blood mini kit), and the concentration of the DNA samples were measured by Qubit dsDNA assay. The gDNA quality was then assessed to make sure A260/A280 is within the range of 1.8 to 2.0. Shearing fragmentation by sonication (covaris M220) was then conducted, followed by end repair, phosphorylation and adaptor ligation. Fragments of size 200–400 bp were selected by bead (Agencourt AMPure XP Kit), followed by hybridization with the capture probes baits, hybrid selection with magnetic beads, and PCR amplification. A bioanalyzer high sensitivity DNA assay was then used to assess the quality and size range. Indexed samples were pooled to be loaded onto the flow cells for sequencing on a Miseq (Illumina, Inc., USA) with 150-cycle pair-end reads.

### Sequence data analysis

Sequence data were mapped to the human genome (hg19) using BWA aligner 0.7.10. PCR duplicate reads were removed before base substitution detection. Local alignment optimization and variant calling and annotation were performed using GATK 3.2. DNA translocation analysis was performed using both Tophat2 and Factera 1.4.3.

### Statistical analysis

Analysis was performed for patients’ characteristics between the mutation positive group and negative group and the percentage of mutation carriers in FNMTC group was compared with that of SNMTC control. Proportions were compared using Fisher’s exact test or Chi-square test, and continuous variables were compared using Student’s t-test or Mann Whitney U-test, as appropriate. A p-value < 0.05 was considered statistically significant.

## Additional Information

**How to cite this article**: Yu, Y. *et al.* Targeted DNA Sequencing Detects Mutations Related to Susceptibility among Familial Non-medullary Thyroid Cancer. *Sci. Rep.* **5**, 16129; doi: 10.1038/srep16129 (2015).

## Supplementary Material

Supplementary Information

Supplementary DataSet 1

## Figures and Tables

**Table 1:** List of the 45 high-confidence non-synonymous mutations identified among 63 NMTC patients.

Family	Sample	Gene	Type	AA change	AF	GVGD	Clin-Var	ExAC ALL	ExAC EAS	SIFT_ score	Polyphen2 _HDIV _score	LRT_ score	Mutation Taster_ score	Mutation Assessor_ score	FATHMM_ score	Radial SVM_ score	LR_ score	Score
BN01	BN001	APC	SNV	L292F	45.3%	Class C0	NA	.	.	0.18	0.468	0.04	0.715	0.69	−2.53	0.136	0.62	3
BN01	BN001	EPCAM	SNV	T164I	48.3%	Class C0	US	0	0	0.09	0.578	0.006	0.999	1.15	−0.66	−0.891	0.143	0
BN01	BN002	APC	SNV	L292F	48.5%	Class C0	NA	.	.	0.18	0.468	0.04	0.715	0.69	−2.53	0.136	0.62	3
BN01	BN002	MSH6	SNV	L1081F	49.4%	Class C0	NA	.	.	0.72	0.045	0.007	1	0.255	−2.17	−0.918	0.291	2
BN03	BN010	BRAF	SNV	D22N	48.9%	Class C0	US	0.0001	0	0.2	0.805	0.304	0.997	0	−1.04	−0.703	0.315	1
BN03	BN049	BRAF	SNV	D22N	49.8%	Class C0	US	0.0001	0	0.2	0.805	0.304	0.997	0	−1.04	−0.703	0.315	1
BN04	BN013	ATM	SNV	C693Y	45.9%	Class C0	NA	.	.	1	0	0.541	1	−0.755	−0.59	−0.941	0.082	0
BN05	BN017	APC	SNV	V1352A	43.4%	Class C0	US	0	0.0005	.	0.595	0.002	1	0.975	−2.5	−0.25	0.483	2
BN06	BN023	MSH6	INDEL	L1358fs	50.6%	NA	LB	0.0023	0.032	.	.	.	.	.	.	.	.	0
BN06	BN024	ATM	SNV	R2691C	47.8%	Class C45	US	0.0001	0.0007	0.02	0.999	0.003	1	1.975	−1.21	0.121	0.552	6
BN09	BN034	MEN1	SNV	A216V	46.5%	Class C0	NA	.	.	0.23	0.053	0.3	1	0.805	−5.8	0.824	0.897	3
BN09	BN035	BRCA2	SNV	V2503I	47.7%	Class C0	US	0	0	1	0.001	0.002	1	−1.845	−1.84	−0.728	0.12	1
BN10	BN036	GNAS	SNV	P220A	45.2%	Class C0	NA	.	.	0.15	0.005	0.002	1	0.695	−3.65	−0.328	0.677	3
BN10	BN036	MSH6	SNV	G355S	48.7%	Class C0	US	0.0001	0.0012	0.69	0.015	0.042	1	0	−1.81	−0.815	0.309	2
BN10	BN038	MSH6	SNV	G355S	51.1%	Class C0	US	0.0001	0.0012	0.69	0.015	0.042	1	0	−1.81	−0.815	0.309	2
BN11	BN039	MSH2	SNV	L719F	47.8%	Class C0	NA	.	.	0	0.974	0.001	1	1.775	−2.01	0.118	0.608	7
BN11	BN040	MSH2	SNV	L719F	49.5%	Class C0	NA	.	.	0	0.974	0.001	1	1.775	−2.01	0.118	0.608	7
BN12	BN043	BRCA1	SNV	R762S	49.8%	Class C0	US	0.0001	0.0015	0	0.993	0.003	1	2.94	−1.09	−0.225	0.61	4
BN13	BN069	EPCAM	SNV	R338G	44.6%	Class C0	NA	.	.	0.01	0.999	0	0.905	2.015	−0.83	0.007	0.504	7
BN13	BN070	MSH6	SNV	K854M	46.4%	Class C65	US	0.0004	0.0015	0	1	0	1	2.71	−2.83	0.822	0.845	7
BN15	BN053	GNAS	SNV	R16C	51.0%	Class C0	NA	0.0001	0.0022	0.01	0.916	.	1	1.39	−2.42	0.042	0.482	4
BN15	BN053	MEN1	SNV	G508D	56.0%	Class C0	US	0.0005	0.0073	0.55	0.059	0.527	1	0	−5.73	0.53	0.844	3
BN15	BN054	MEN1	SNV	G508D	55.9%	Class C1	US	0.0005	0.0073	0.55	0.059	0.527	1	0	−5.73	0.53	0.844	3
BN16	BN052	MSH6	INDEL	NA	54.0%	NA	US	0.0013	0.0042	.	.	.	.	.	.	.	.	0
BN17	BN081	BRCA1	INDEL	SS955S	49.6%	NA	NA	.	.	.	.	.	.	.	.	.	.	0
BN17	BN081	WRN	SNV	V755A	52.3%	Class C0	NA	.	.	0.33	0.212	0.035	0.991	1.15	3.61	−0.956	0.012	0
BN17	BN082	BRCA1	INDEL	SS955S	50.0%	NA	NA	.	.	.	.	.	.	.	.	.	.	0
BN18	BN084	BRCA2	SNV	F3328C	48.7%	Class C15	NA	0	0	0.09	0.89	0.016	1	1.79	5.85	−0.876	0.004	0
BN19	BN085	BRCA2	SNV	G2508S	48.2%	Class C55	US	0.0001	0.0015	0.05	1	0	1	2.71	−2.19	0.748	0.819	8
BN19	BN086	APC	SNV	A2778S	44.9%	Class C0	US	0.0002	0.0023	.	1	0	1	0.975	−1.53	−0.021	0.573	5
BN19	BN086	BRCA2	SNV	G2508S	44.2%	Class C55	US	0.0001	0.0015	0.05	1	0	1	2.71	−2.19	0.748	0.819	8
BN19	BN087	APC	SNV	A2778S	49.8%	Class C1	US	0.0002	0.0023	.	1	0	1	0.975	−1.53	−0.021	0.573	5
BN21	BN094	TIMM44	SNV	R277Q	54.3%	Class C0	NA	0	0	0.01	0.997	0	1	3.155	−1.66	0.572	0.679	8
BN22	BN095	GNAS	INDEL	P459PDAPADPDSGAAR	57.9%	NA	NA	.	.	.	.	.	.	.	.	.	.	0
BN22	BN095	MSH6	SNV	A36V	55.4%	Class C0	US	0.0002	0.0008	0.2	0.386	0.001	0.999	0.55	−2.02	−0.514	0.411	1
BN22	BN096	GNAS	INDEL	P459PDAPADPDSGAAR	60.0%	NA	NA	.	.	.	.	.	.	.	.	.	.	0
BN22	BN096	MSH6	SNV	A36V	51.1%	Class C0	US	0.0002	0.0008	0.2	0.386	0.001	0.999	0.55	−2.02	−0.514	0.411	1
BS01	BS001	MSH2	SNV	M261V	43.5%	Class C0	US	.	.	1	0.097	0	1	0.805	−2.32	−0.748	0.334	3
BS02	BS002	MSH2	SNV	V78I	50.3%	Class C25	NA	0	0.0002	0.03	0.998	0	1	2.38	−2.56	0.783	0.82	8
BS07	BS007	APC	SNV	L662I	53.0%	Class C0	NA	0.0001	0.0008	0	0.995	0	1	2.045	−0.31	−0.372	0.384	5
BS07	BS007	WT1	SNV	S322N	46.6%	Class C0	NA	.	.	0.38	0.001	0.054	1	−0.69	3.41	−1.054	0.039	1
BS12	BS012	MSH6	SNV	A53D	44.0%	Class C0	NA	.	.	0.36	0.739	0.967	1	0.55	−1.89	−0.493	0.425	2
BS12	BS012	MSH6	INDEL	G56GPR	44.7%	Class C0	US	.	.	.	.	.	.	.	.	.	.	0
BS14	BS014	BRCA1	SNV	A1368V	45.4%	Class C0	NA	.	.	0.62	0.985	0.663	0.995	−0.145	−1.71	−1.016	0.042	3
BS17	BS017	ATM	SNV	I346N	49.2%	Class C45	NA	.	.	0	0.938	0	0.999	1.845	−0.35	−0.373	0.311	3

AA: Amino Acid; NA: Not Available; US: Uncertain Significance; LB: Likely Benign. AF: Allele Frequency; Score: Number of algorithms predicting the variant to be “damaging”; BN: FNMTC samples; BS: SNMTC samples; 14 variants with score ≥ 4 were predicted to be damaging or deleterious by at least half of the predicting algorithms; More details are available in Supplementary Table S1 online.

**Table 2:** Association of germline mutations and clinical-pathological characteristics.

	Mutation +	Mutation −	p-value
N	35	26	
Female: Male	28:7	20:6	0.77
Age	43.26 ± 9.66	47.19 ± 13.02	0.18
Tumor size	1.05 ± 0.50	1.01 ± 0.47	0.73
Multifocality	16(45.7%)	10(42.3%)	0.79
Bilaterality	12(34.3%)	8(34.6%)	0.98
Total thyroidectomy	15(42.9%)	12(46.2%)	0.80
Central compartment neck dessection	34(97.1%)	25(96.2%)	0.83
Extrathyroidal extension	16(45.7%)	14(53.9%)	0.53
Central lymph node involvement	24(68.6%)	8(30.8%)	0.003
Lateral lymph node involvement	7(20.0%)	6(23.1%)	0. 77

Proportions were compared using Fisher’s exact test or Chi-square test, and continuous variables were compared using Student’s t-test or Mann Whitney U-test, as appropriate. A p-value < 0.05 was considered statistically significant.
