# Supplementary material for: Targeted DNA Sequencing Detects Mutations Related to Susceptibility among Familial Non-medullary Thyroid Cancer
Source: Sci Rep. 2015 Nov 4;5:16129. doi: 10.1038/srep16129 (PMC4632085; doi:10.1038/srep16129)
Supplement: Supplementary Information [file srep16129-s1.pdf]

Targeted DNA Sequencing Detects Mutations Related to Susceptibility among Familial Non-medullary Thyroid Cancer

Yang Yu[1,3], Li Dong[1,3], Dapeng Li[1,3], Shaokun Chuai[4], Zhigang Wu[4], Xiangqian Zheng[1,3], Yanan Cheng[2,3], Lei Han[2,3], Jinpu Yu[2,3*], Ming Gao[1,3*]

[1]Department of Thyroid and Neck Tumor, Tianjin Medical University Cancer Institute and Hospital, National Clinical Research Center for Cancer, Huanhuxi Road, Ti-Yuan-Bei, Hexi District, Tianjin 300060, China.

[2] Cancer Molecular Diagnosis Core, Tianjin Medical University Cancer Institute and Hospital, National Clinical Research Center for Cancer, Huanhuxi Road, Ti-Yuan-Bei, Hexi District, Tianjin 300060, China.

[3]Key Laboratory of Cancer Prevention and Therapy, Tianjin, Huanhuxi Road, Ti-Yuan-Bei, Hexi District, Tianjin 300060, China.

[4]Guangzhou Burning Rock Biotech, Guangzhou International Biotech Island (GIBI), Guangzhou 510300, China.

Yang Yu and Li Dong contributed equally to this work.

[*]Correspondence and requests for materials should be addressed to Ming Gao (email: gaoming68@aliyun.com) or Jinpu Yu (email: yujinpu@tjmuch.com).

Supplementary Fig. S1

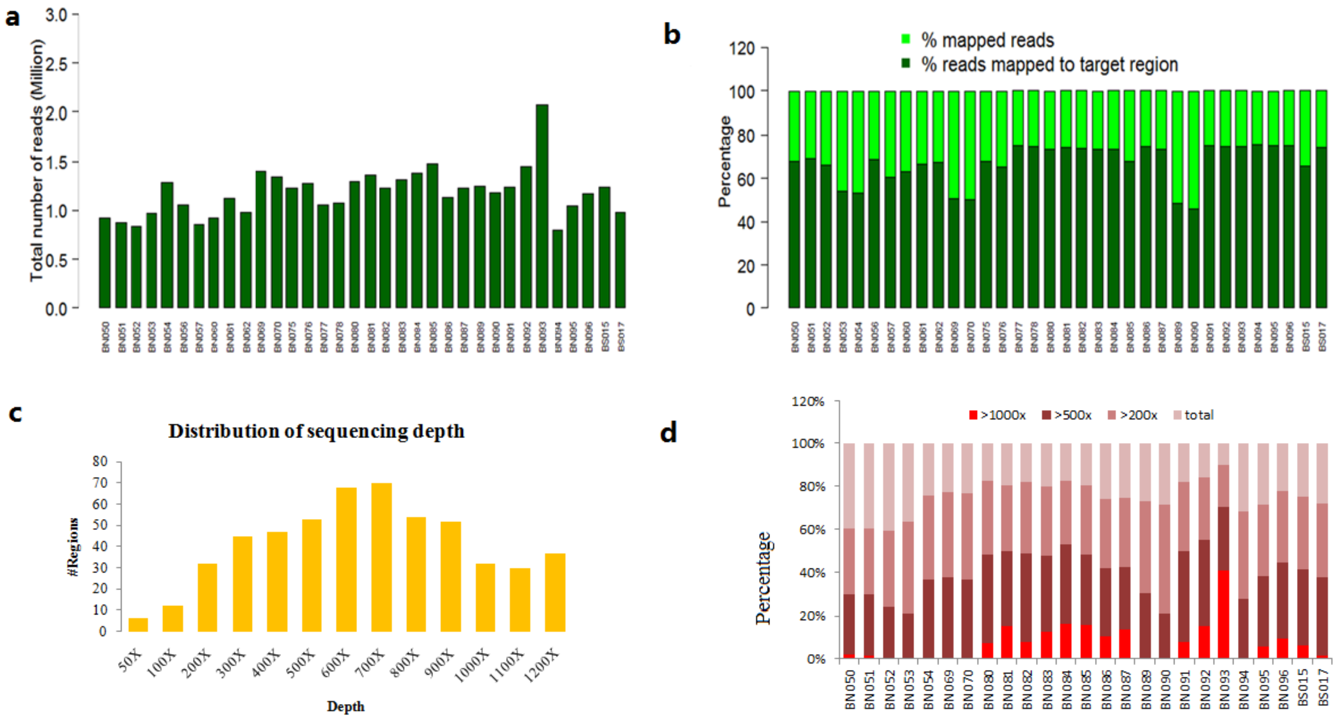

Supplementary Figure S1. Quality assessment of the targeted sequencing data among 25 samples in the second batch. a. Total number of reads in every sample. **b.** Percentage of all mapped reads and percentage of reads mapped to target regions for every sample. **c.** The average distribution of sequencing depth over regions across all samples. **d.** The coverage depth in each sample.

Supplementary Fig. S2

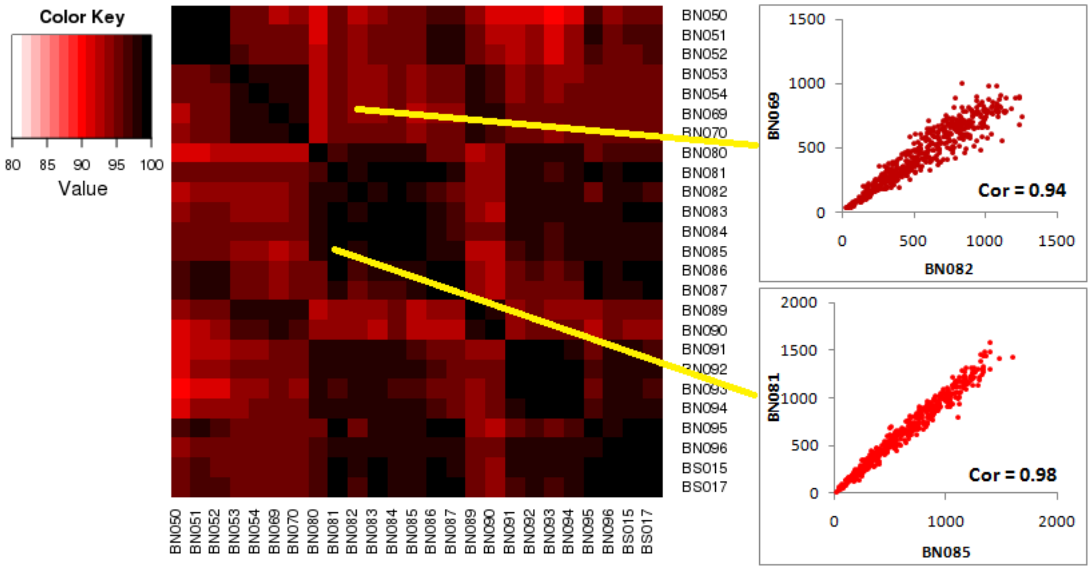

Supplementary Figure S2. The correlation of coverage depth in each target region between 25 samples in second batch. a. A heatmap of such correlation between each pair of samples. **b.** The scatterplot of coverage depth across all regions in the least correlated pair of samples. **c.** The scatterplot of coverage depth across all regions in the most correlated pair of samples

Supplementary Table S1.

In order to display the results of the analysis completely, the detailed information is shown in the excel spreadsheet.

Supplementary Table S1. List of the detailed information about the 45 high-confidence non-synonymous mutations identified among 63 NMTC patients. (Note: AA: Amino Acid; NA: Not Available; US: Uncertain Significance; LB: Likely Benign. AF: Allele Frequency; Score: Number of algorithms predicting the variant to be "damaging"; BN: FNMTC samples; BS: SNMTC samples. Out of all 45 variants detected, 14 variants marked in red in the last column were predicted to be damaging or deleterious by at least half of the predicting algorithms. The bold part represents three matching variants (BRCA2 G2508S, MSH2 L719F, and APC A2778S) within FNMTC families predicting to be damaging.)
